# Supplementary material for: Nutritional Behaviors of Polish Adolescents: Results of the Wise Nutrition—Healthy Generation Project
Source: Nutrients. 2019 Jul 13;11(7):1592. doi: 10.3390/nu11071592 (PMC6682866; doi:10.3390/nu11071592)
Supplement: Supplementary file 1 [file nutrients-11-01592-s001.pdf]

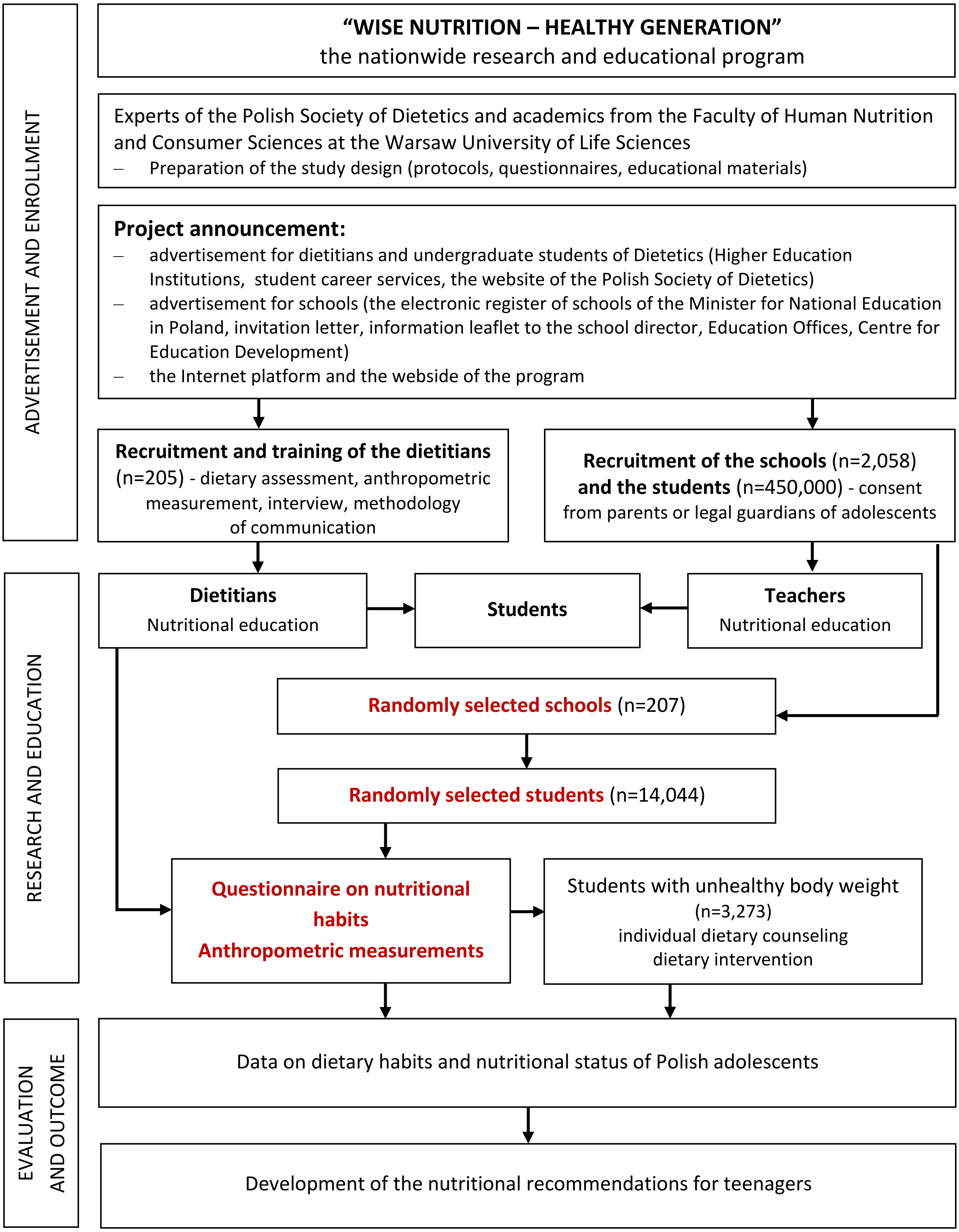

Figure 1. The Wise Nutrition - Healthy Generation project diagram (the part of the program presented in the article is marked in red).
